# Surgery of Primary Melanomas

**DOI:** 10.3390/cancers2020824

**Published:** 2010-05-11

**Authors:** Piotr Rutkowski, Marcin Zdzienicki, Zbigniew I. Nowecki, Alexander C. J. van Akkooi

**Affiliations:** 1 Soft Tissue/Bone Sarcoma and Melanoma Department, M. Sklodowska-Curie Memorial Cancer Center and Institute of Oncology, Warsaw, Poland; E-Mails: mzdzienicki@coi.waw.pl (M.Z.); nowecki@coi.waw.pl (Z.I.N.); 2 Erasmus University Medical Center–Daniel den Hoed Cancer Center, Rotterdam, The Netherlands; E-Mail: a.vanakkooi@erasmusmc.nl (A.C.J.v.A.)

**Keywords:** cutaneous melanoma, melanoma treatment, surgery, wide excision

## Abstract

Surgery remains the mainstay of melanoma therapy, regardless of the tumor site. Only the early diagnosis combined with proper surgical therapy currently gives patients affected by this malignancy the chance for a full cure. The main goal of surgical therapy is to provide the local control of the disease and to secure long-term survival of the patient without reasonable functional and esthetic impairment. The recommended method of biopsy—excisional biopsy, as an initial diagnostic and, to some extent, therapeutic procedure—is performed under local anesthesia as an elliptical incision with visual clear margins of 1–3 mm and with some mm of subcutaneous tissue. The extent of radical excision of the primary tumor (or scar after excisional biopsy) is based on the histopathologic characteristics of the primary tumor and usually consists of 1–2 cm margins with primary closure. The philosophy behind conducted randomized clinical trials has been to find the most conservative surgical approach that is able to guarantee the same results as more demolitive treatment. This has been the background of the trials designed to define the correct margins of excision around a primary cutaneous melanoma. Much less definition can be dedicated to the surgical management of patients with non-cutaneous melanomas.

## 1. Introduction

Melanoma is a malignant tumor arising from melanocytes. The most common localization of the tumor is the skin, which includes about 95% of cases. But melanomas can be observed in other locations such as the mucosal membranes of gastrointestinal tract and genitourinary system or in the eye. The incidence of melanoma varies in different populations and depends on some biological (race, skin type, number of nevi or presence of dysplastic nevi), lifestyle and/or environmental (sun or tanning beds exposure, geographical location) factors. The highest incidence rate can be observed in Caucasian inhabitants of Australia (42.9 per 100,000 women and 55.8 per 100,000 men). European countries and the United States register incidence on a medium level (from seven to 20 cases per 100,000 per year). The incidence of and mortality resulting from melanoma is increasing worldwide. The estimation for the Caucasian population from different countries shows an annual 3–7% increase of incidence with a doubling of incidence every 10–20 years [1,2,3,4,5,6]. 

The prognosis after melanoma diagnosis depends on parameters of the primary tumor. The main prognostic factors are: the Breslow thickness of the primary tumor, the presence of ulceration and the mitotic rate. The results of treatment have improved during the last decades (from overall survival of 23–35% in the middle of the twentieth century to about 78% at the end of it). These results are due to an increase in early diagnosis rather than from achievements in melanoma treatment [7,8]. 

Surgery remains the mainstay of melanoma therapy of any primary site and only early diagnosis combined with proper surgical therapy currently gives the chance for cure of patients affected by this malignant tumor. The main goal of surgical therapy is to provide the local control of the disease and to secure long-term survival of the patient without reasonable functional and esthetic impairment. Currently, appropriate surgery is dependent upon melanoma staging. The limited efficacy of systemic and adjuvant treatment for metastatic melanoma emphasizes the importance of effective initial surgical therapy. Appropriate surgery of the primary lesion allows achieving durable local control and may be curative for patients without metastases. There are two major issues that should be considered regarding surgery of the primary tumor: properly performed diagnostic biopsy and radical excision of the primary tumor (or excision of the scar after excisional biopsy). On the other hand, surgical approaches should be tailored to the individual clinical situation.

## 2. The Diagnosis of the Melanoma (Excisional Biopsy)

The excisional biopsy of the suspicious nevi or skin tumor is the widely accepted first step in melanoma management, leading to its diagnosis. The diagnosis of melanoma can be suspected based on well-known rules of the ABCD/E system (A–asymmetry, B–irregular borders, C–color changes, D–diameter >5 mm, and E–elevation), or the Glasgow system and depends on detailed physical examination of the entire skin. In early lesions (diameter ≤5 mm or slightly raised), the clinical diagnosis can be significantly improved by non-invasive epiluminescence microscopy (dermoscopy) [9,10,11].

**Figure 1:**
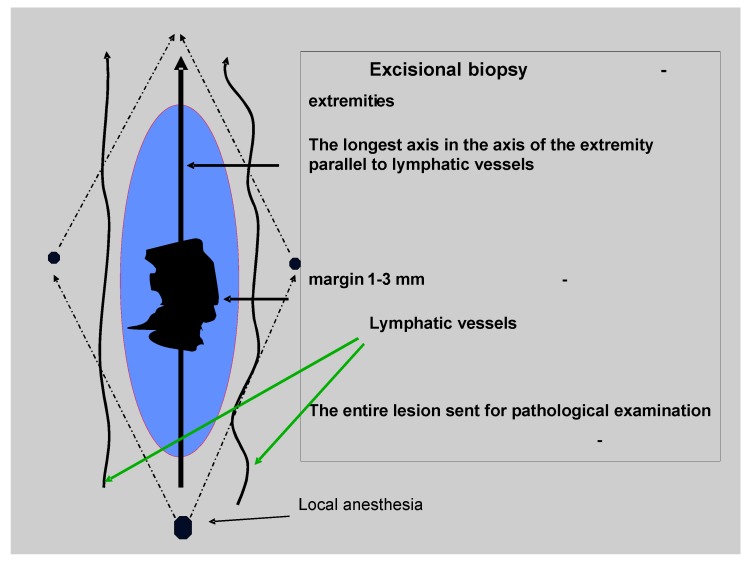
Practical considerations of excisional biopsy of a skin lesion suspicious of being melanoma (courtesy of W. Ruka) [15].

The technique of excisional biopsy is simple. The suspected lesion should be resected with the minimal margin of normal skin being 1–3 mm wide and with 2 mm margin of subcutaneous tissue (Figure 1). Typically this procedure is performed under local anesthesia and it is not necessary to hospitalize a patient. The direction of the surgical incision should be compatible with lymphatic drainage of the surrounding skin: on the trunk it should be oriented towards the nearest lymphatic basin and on the extremities along the long axis of the limb. The biopsy is essential for diagnosis and full microstaging of the primary tumor (pT), which determines the choice of further therapy and establishes basic prognostic factors. The pathologic report should, at minimum, include the Breslow thickness (mm), presence or absence of ulceration and mitotic rate (histologically defined as mitoses/mm^2^), status of resection margin, and presence of satellitosis [12].

In rare cases of large lesions that are difficult to remove under local anesthesia or for lesions situated in certain anatomic areas (*i.e.*, large lentigo melanoma, subungual melanoma, *etc.*), where the skin sparing is important, incisional or punch biopsy may be an acceptable alternative [13]. It is currently believed that this procedure does not increase the risk of metastasis and has no adverse effect on the final prognosis if subsequent radical surgery is performed within 4–6 weeks [14]. Shave or curette-type biopsies should not be performed on skin lesions suspected of melanoma, because these techniques limit the amount and quality of specimen for pathological assessment. 

The initial biopsy should not compromise, by flap transfer or transverse orientation of the incision to the long axis of the extremity, a subsequent sentinel node biopsy in a patient who can be eligible for this procedure.

## 3. The Radical Surgery of the Primary Tumor

After pathological confirmation of melanoma, the remaining skin scar should be re-excised. This resection is, in fact, a radical therapeutic procedure, aimed to prevent the local recurrence and in cases without micrometastatic disease - to cure the patient. The extent of excision is based on the hypothesis that the incidence and the extent of local recurrences may be predicted by histopathologic characteristics of the primary tumor, and the most important predictive features are thought to be tumor thickness and ulceration. For a long time, the sufficient margins of resection have been a matter of discussion. Until the 1970s, the standards of care were margins of radical excision ranging from 3 to more than 5 cm. However, accumulating evidence from controlled clinical studies over the past three decades has limited definitive resection margins around primary melanoma site to 1-2 cm instead of these 3–5 cm margins. These trials showed that narrower resection margins do not have any negative influence on patients’ survival and the rate of metastatic disease. Analysis of overall survival in randomized trials shows equal prognosis for melanomas with narrow and for patients with wide resection margins. The rationale for wider excision was based on the observation that the risk of local recurrences correlated with increasing melanoma thickness. However, it has been shown that most of locally recurrent melanoma cases demonstrate histological features of metastasis rather than those of residual, incompletely or “inadequately” excised primary melanoma [15,16,17]. This implies that the local recurrence process is in the majority beyond surgical control, because it is related to biological aggressive behavior of the primary tumor (which depends mostly on the tumor thickness and ulceration) manifesting as vascular dissemination of melanoma cells. The presence of microsatellites suggests aggressiveness of melanoma and correlates with dismal prognosis similar to that for patients with regional nodal metastases [18,19,20,21], therefore wider margins of surgical resection are unlikely to cure this group of patients. On the other side, it is unquestionable that inadequate margins can increase the risk of locoregional recurrences and may be associated with decreased overall survival. Hence, at present the philosophy of recommendations based on clinical trials performed during the last three decades is finding the most conservative surgical approach giving the same effective results as more radical procedure. 

Six randomized, prospective trials (Table 1) have evaluated the effect of the width of definitive excision margins according to the thickness of the primary lesion on the patients’ survival and melanoma recurrences [15,16,17,18,19,20]. No clinical study has shown a survival disadvantage for narrower *versus* wider radial excision margins for melanomas of any thickness and all studies yield similar conclusions: that an excision of melanoma with conservative surgical margins ensures durable control of the disease similar to those observed with more extensive resections. 

In 1988, the WHO Melanoma Group Trial No. 10 published the results of the first randomized trial planned for resolving the problem of sufficient margins of primary resection. In the trial, 1 cm wide margin were compared with 3 cm ones in 612 patients with ≤2mm thick melanoma. The analysis of collected data did not show any benefit for wider resectioning [22]. Those results for thin melanomas (≤2 mm) have been later confirmed by the Swedish Melanoma Group (comparing a 2 cm to 5 cm margin in 989 patients) and the French Cooperative Group (also comparing 2 cm to 5 cm margins [23,24,25]. In the French trial, the 10-year disease-free survival rates were 85% for the group with a 2-cm margin and 83% for the group with a 5-cm margin. There was no difference in the 10-year overall survival rates (87% *vs*. 86%, respectively). The long term follow up results of the Swedish trial also showed no significant difference with respect to recurrence-free or overall survival between the groups. The issue in patients with thicker melanomas has been evaluated by the Intergroup Melanoma Surgical Trial and UK Melanoma Study Group. The Intergroup Melanoma Surgical Trial did not show any significant difference between 2 cm margins compared to 4 cm in 486 patients with 1–4 mm thick melanomas. The local recurrence rate and overall survival were similar in both groups [26]. In only 11% of the group of melanoma patients treated with 2-cm margins, the use of a skin graft became necessary as compared to 46% of cases in the group using 4-cm wide excision margins (P < 0.001). The data collected by the UK Melanoma Study Group from 900 patients with melanomas ≥2 mm suggested better locoregional disease control for 3 cm wide margin radical resection in comparison with 1 cm margin. Overall survival was similar in both groups [27]. 

**Table 1:** Clinical trials evaluating the width of radical excision surgical margins for primary melanoma.

Breslow thickness [mm]	Clinical study	Patients(No.)	Margins [cm]	Overall survival (at years)	Ref.
	French Cooperative Group	336	2 *vs*. 5	No difference(10-year)	[25,28]
≤2	Swedish Melanoma Group	989	2 *vs*. 5	No difference(5-year)	[23,29]
	WHO Melanoma Group Trial No. 10	612	1 *vs*. 3	No difference(10-year)	[30,31]
1–4	Intergroup Melanoma Surgical Trial	486	2 *vs*. 4	No difference(6-year)	[26,32,33]
≥2	UK Melanoma Study Group	900	1 *vs*. 3	Not reported; hazard ratio for death was similar in both groups (5-year)	[27]
	Swedish Melanoma Trial Group	1,000	2 *vs*. 4	Final results not reported; preliminary results indicated no difference (5-year)	[34]

For melanomas thicker than 4mm there is no data from randomized trials indicating the width of sufficient margin of resection. However, a retrospective analysis covering 278 patients with melanomas ≥4 mm tumor thickness was not able to show any benefit for margins wider than 2 cm in terms of local recurrence, disease-free survival, and overall survival [35]. This study also supported the hypothesis that recurrences are related to the biological aggressiveness of primary melanoma, because overall survival rate after locoregional recurrence was lower in the group with 3 cm margins of excision than in the group with 1 cm margins of excision: 18.5 months *vs.* 27.9 months, respectively. Because locoregional recurrence rates in this trial were higher in the 1-cm than 3-cm group, the authors recommended margins of excision wider than 1 cm for melanomas thicker than 2 mm. However, this statistical significance was established only when local and regional nodal recurrences were added together, and we do not know if the current approach with a sentinel node biopsy would change these results. The possibility of uniform recommendations for all melanomas thicker than 2 mm was provided in the most recent trial: a Scandinavian-Baltic trial randomizing 936 patients with primary cutaneous melanomas with a tumor thickness above 2.0 mm (pT3, pT4) to the treatment with definitive margin of 2 cm or margin of 4 cm. Preliminary results (final results are still pending) presented during Sixth Word Congress on Melanoma [34] indicate no difference between these two analyzed groups in term of treatment outcomes.

Taking all these trials into account, it can be reasonable to conclude that 2 cm margin can be considered appropriate for virtually all melanomas thicker than 2 mm. For melanomas no larger than 1 mm, 1-cm margins of excision are sufficient. Data about invasive melanoma 1 to 2 mm thick are inconclusive, because trials comparing 1-cm and 2-cm margins probably will never be performed. However, many national recommendations agree that 1 cm margins may be sufficient and safe, even in melanomas thicker than 1 mm (supported by the results of the WHO trial), especially in regions of anatomic constraints associated with expected functional or cosmetic deformities (e.g., the face, distal part of limbs). Limiting resection margins in melanoma provides the possibility of primary closure of the wound in most cases with a reduced need for skin grafts or complex tissue closures. Moreover, this limits the costs and morbidity per patient, providing better functional and esthetic outcomes as well as offering statistically identical outcomes in terms of survival and recurrences as wider excisions. Of course, there are some subgroups of patients for which the margin recommendations are based on opinions of experts only, as well as margins that may be modified to adapt to the individual anatomic or cosmetic circumstances. In the cohort of patients with melanoma *in situ*, a margin of 5 mm is recommended, because these lesions have no risk for metastases, but they are at risk for local recurrences. However, there are some data that for *in situ* melanomas [36,37,38], wider margins (1 cm) may be justified.

Desmoplastic melanomas have been associated with worse prognosis and wider margins have been recommended, however the large report from the Sydney Melanoma Unit did not confirm that this entity is prognostically poorer [39].

The excision should include the subcutaneous tissue up to the fascia. Depth of the excision has never been explored in current prospective clinical trials. However, there is no evidence of better treatment results after resection of the underlying muscle fascia [40,41,42]. 

From the technical point of view, the radical treatment of the primary melanoma can be divided into two main elements: the first is the margins and the orientation of definitive excision, the second is the reconstruction of the defect. It is necessary to remember that, in fact, in majority of cases radical surgical treatment of primary melanomas consists of the excision of the scar after excisional biopsy to the margins established on the basis of the primary tumor thickness, established by the excisional biopsy. We previously discussed the margins of the definitive excision, which should be measured in each direction from periphery of the lesion or scar. The long axis of the incision should be in the direction of the lymphatic drainage and parallel to the long axis of the extremity, which decreases the level of peripheral edema (especially in cases of subsequent inguinal lymph node dissection). Primary closure usually requires that the longest axis of an elliptical incision be at least three times longer than the short axis. Excision should include also subcutaneous tissue down to, but not routinely including, the underlying fascia. The majority of wounds after 1–2 cm margins of excision can be closed by primary sutures. Skin flaps are undermining in the plane to alleviate tension and to permit approximation of wound borders. In the minority of cases, when the wound cannot be closed primarily, more complex reconstruction techniques are employed, such as skin grafting and use of local and distant flaps. Free skin grafts are recommended if the higher possibility of local recurrences/in-transit metastases exists, because they make it easier to check for possible recurrences (as compared to the flaps). The skin graft donor site should be outside the area of potential in-transit metastases (e.g., contralateral thigh). The full-thickness grafts are used commonly on the face or hands for better esthetic and cosmetic results and may be taken from behind the ear, from the supraclavicular region or from the site of sentinel node biopsy. The role of Mohs’ micrographic surgery is controversial and generally is not recommended for invasive lesions. The hypothetic risk of cutting part of the melanoma during attempts to excise clear margins during Mohs’ surgery is the reason why this technique is not accepted by most oncology centers. However, it seems that this technique may have some role in the management of melanomas in anatomic sites where standard margins may prove difficult to achieve.

Typically, the radical operation of primary tumor is followed by the sentinel lymph node biopsy (SLNB) procedure. The indication is the presence of primary melanoma without clinical, radiological or histological evidence of metastases. Minimal requirements for SLNB on melanoma patients have not been reached; some authors suggest the Breslow thickness of 0.75 mm and others 1 mm. In 2002, following the introduction of the sentinel node procedure, a new staging classification was introduced, the cut point between a very low risk of metastases and a higher risk changed from a 0.76 mm to 1.00 mm thickness [19,43,44,45]. Currently, the most common indication for application of SLNB is primary cutaneous melanoma with Breslow thickness ≥1 mm or ulcerated or Clark level ≥IV or mitotic index ≥1/mm^2^. Several studies have already proven that SLNB offers several benefits in the course of melanoma patient management: better staging, avoiding unnecessary ELND, excellent prognostic information, facilitation of therapeutic lymphadenectomy, homogeneity of patient populations in clinical trials on adjuvant therapy, and—from the patient’s point of view—increased sense of safety and accuracy of care. At the moment, the SLNB is widely recommended as a staging procedure, however its influence on overall survival of melanoma patients remains unclear [46,47]. In the case of positive SLNB, radical lymph node dissection is recommended.

SLNB is a surgical procedure, connected with some risk of complications and relatively expensive. In some centers, before the SLNB era, the regional lymphatic basin was evaluated with non invasive methods, which may reveal early lymph node metastases and allowed avoiding surgical biopsy. To the present date, the ultrasound examination, combined with US-guided fine needle biopsy, seems to be the most promising method. Using this technique, up to 65% of SLNB could be avoided [48,49].

In some centers, FDG-PET scans have been evaluated for suitability in early detection of melanoma metastases. However, the analysis of acquired data showed some effectiveness of the method for diagnosis of distant melanoma metastases, but not micrometastases in regional lymphatic basins, and this method currently cannot replace SLNB [50,51]. 

There is no standard adjuvant treatment for high risk melanoma patients after the radical resection of the primary tumor, but the results of surgical treatment alone are still unsatisfactory. In the past decades, many schemes of systemic complimentary treatment, including chemotherapy, immunotherapy or both, have been evaluated. Most of those therapies did not improve the overall survival of high risk melanoma patients. One exception is interferon. Several studies showed benefits in term of improvement in relapse-free survival for patients treated with adjuvant interferon. A large meta-analysis showed that interferon-therapy improved the overall survival in 3% of the general studied melanoma population, but the prolongation of disease free survival with interferon treatment is more evident—up to 7% [52,53,54,55]. 

To improve the results of surgical treatment of the primary melanoma in term of loco-regional recurrences in the limb, the use of prophylactic isolated limb perfusion (ILP) with melphalan has been proposed. Although the first retrospective studies showed an improved outcome for patients with a high-risk primary melanoma after ILP treatment [56,57], a large prospective randomized study with 832 patients [58] did not demonstrate any benefit of prophylactic ILP on the time to systemic metastases or on overall survival and suggested improvement of locoregional control only. Thus, as the procedure is costly and accompanied by considerable morbidity, prophylactic ILP cannot be recommended as adjuvant therapy after surgical excision of high-risk primary melanoma.

## 4. Primary Melanoma of Specific Sites

### 4.1. Melanoma of Mucosal Surfaces

The primary melanoma located on mucosal surfaces is a very rare disease that represents less than 3% of all melanoma [59] and has biologically aggressive behavior. Among mucosal sites, the most frequent are: head and neck mucosal membranes, female genital tract (mostly vulva), and anorectal region (all for approximately 30%) [60,61]. The rarest are primary melanomas originating from urinary tract sites and the stomach/bowel. Early detection is difficult for these tumors because of the occult anatomic locations.

The diagnosis must be established after a full thickness biopsy of the suspicious lesion, with the exception of small lesions suitable for excisional biopsy. Incisional biopsy should include a representative sample from the border of the lesion to help the pathologist in differentiating a primary mucosal melanoma from mucosal melanoma metastasis. Clinical research in mucosal melanoma is necessary, because less than 20% of patients are alive five years from the date of initial diagnosis. The general therapeutic consensus is to attempt a complete surgical excision of the primary site with clear margins followed by postoperative radiation therapy for microscopic or macroscopic residual disease or nodal involvement.

The head and neck mucosal melanoma affects mainly the nasal and oral cavity. The primary approach for treatment of mucosal melanoma is a wide surgical resection, but the results of the treatment are very disappointing. The five-year overall survival remains in the range of 13–34% [62,63,64,65]. While many cases of mucosal melanoma are treated with surgery alone, radiotherapy or chemotherapy as an adjuvant therapy or even the only modality (radiotherapy) is employed more frequently than in cutaneous melanoma [66].

The most frequent primary site of genital melanoma is the vulva [67,68,69,70]. Similar to other mucosal melanoma, it shows a tendency for a high incidence of local recurrences and distant metastases (hematogenous and lymphogenous). Multiple studies of more than 350 cases of vulvar melanoma suggested that radical vulvectomy (with or without inguinofemoral lymphadenectomy) does not improve the overall and disease-free survival compared to more limited resection (wide local excision or partial vulvectomy with adequate tumor-free margins) [71,72,73,74]. Radical vulvectomy, in contrast to wide local excision, is associated with higher morbidity, including lymphedema and abandonment of sexual intercourse after surgery in most cases, as well as the wound breakdown, cystocele, rectocele, urinary incontinence, vaginal stricture and dyspareunia. Consequently, the conservative treatment with a wide local excision has been proven to be as effective as vulvectomy for overall, tumor-specific survival. The width of the tumor-free margins for excision of vulvar melanoma is rarely defined, and it is difficult to analyze statistically. Most cases of melanoma of the penis have reported amputation of the organ [75]. In genital melanomas, staging procedure with sentinel node biopsy could be considered.

The majority of melanoma of the anorectal region arises below the dentate line in the squamous mucosa, and, similar to other mucosal melanomas, this occult localization contributes to delayed diagnosis in advanced stages. The mortality rate is high and no significant differences between abdominoperineal resection and local excision—both in overall and disease-free survival—have been found [76,77]. Consequently, most studies suggest that extended procedures add little value. A limited approach includes local excision potentially combined with the inguinal lymph node dissection and local tumor destruction by cryosurgery or fulguration. The procedure of choice is a wide excision with clear margins (ultrasound can be helpful in delineating lesions) that avoids permanent colostomy. Abdominoperineal resection should be attempted only for large primary tumors not amenable for local excision and for local recurrences with documented distant dissemination. The role of radiotherapy is under investigation.

### 4.2. Subungual Melanoma of the Hand or Foot

Subungual melanomas account for less than 1% of entries in the databases of tertiary referral centers. One of the major problems is delayed diagnosis that leads to presentation at a more advanced stage. Traditional recommendations advocate amputation of the affected finger or toe. Recent studies suggest more distal, function-preserving amputations without compromising the survival and recurrence rates. One study from 2007 has proposed even more conservative, local excision with reconstruction using a local flap to minimize disability. The fingers other than thumb require amputation at the distal interphalangeal joint, but toes can be amputated at the metatarsopharyngeal joint with no functional deficit, unless in the hallux [78,79,80].

### 4.3. Melanoma of the Face and Scalp

For melanomas of the face, narrower margins of excision (1 cm or lower) are generally accepted, although no formal prospective study has been performed. This recommendation is guided by difficulties related to potential damage of surrounding structures (*i.e.*, eyelids, nostrils and mouth) by the closure or subsequent scarring [81]. Melanoma of the ear is treated by the wedge excision or partial amputation [82]. 

The most common pathological type on the face of elderly patients is lentigo malignant melanoma (LMM). Due to the relatively large size of LMM, it is very important to establish obvious clinical borders (by visual inspection, dermoscopy or illumination under the Wood’s light). 

The alternative therapeutic option offering good cosmetic results for elderly patients with LMM is radiotherapy. Multi-fractionated regimes using 7 mm to 10 mm margins have been effective for treatment with recurrence rates of approximately 7% [83,84,85,86]. A recent retrospective study on 117 cases of LM and LMM treated with a staged, margin-controlled excision technique found that a mean total surgical margin required for excision of LMM was 10.3 mm. [87].

In some oncology centers there are attempts to apply the Mohs micrographic surgery (MMS) in primary melanoma treatment of the head and neck region. Those attempts are usually limited to “difficult” localization of the tumor, in which conserving the surrounding skin is essential. Although some authors reported good results of such procedures, the role of MMS in melanoma treatment is still controversial and is not recommended [38,88,89,90]. 

## 5. Ocular Melanoma

Ocular melanomas are mainly represented by uveal melanomas, which constitute the most common adult primary intraocular malignancy and second most frequent type of primary melanoma. Pathogenesis, histology, natural history, and metastatic pattern (hematogenous, mainly to the liver) of uveal melanoma are completely different from cutaneous or mucous membrane melanoma. Uveal melanoma has no natural barriers to its deep invasion due to the lack of uveal basal membrane. It is classified as a tumor arising from the choroid, ciliary body or iris. In contrast to its cutaneous counterpart, uveal melanoma is diagnosed not by biopsy but mainly by using non-invasive techniques (ophthalmoscopy, fluorescein angiography, ultrasound). In the past, enucleation was the treatment of choice; however, recent advances have allowed, in many cases, for preservation of the eye and vision residual functions without compromising the control of the tumor. A study that performed pre-enucleation irradiation based on a theoretical assumption that the treatment would reduce local and hematogenous dissemination reported a small but statistically insignificant advantage of radiotherapy (five-year overall survival of 62% *vs*. 57%). Alternatives to enucleation include various types of radiotherapy (external beam charged particle irradiation with protons or helium ions, radioactive plaque brachytherapy), local trans-scleral or transretinal tumor resection, diode laser phototherapy, and transpupillary thermotherapy [91]. The Collaborative Ocular Melanoma Study (COMS) has evaluated different methods of treatment in groups of patients with small tumors (less than 3 mm in elevation), medium tumors (3–10 mm in height and 15 mm in diameter; enucleation *vs*. brachytherapy), and large tumors (greater than 10 mm in thickness and 16 mm in basal diameter; preoperative adjuvant radiotherapy or surgery only). The experience from this and other studies suggests similar outcomes for different methods of local treatment. However, the major impact on uveal melanoma therapy may be related to new methods of control of metastatic disease [92,93,94,95,96,97].

## 6. Final Considerations

The adequacy of a surgical treatment on melanoma patients is the most important milestone in the natural history of the disease, once the diagnosis has been confirmed. Surgery presents a fundamental role in the initial stages of the disease. The excision of a primary melanoma is generally considered a minor surgical procedure. The most important trials conducted were planned to define whether the indication towards the correct resection margin around a primary melanoma could be as limited to 1–2 cm instead of 3–5 cm, as accepted for many decades as the gold standard approach to excise a primary melanoma. All these studies confirmed that the more conservative approach is effective in terms of overall survival and local control. However, they did not create an univocal approach on some primary tumor thickness ranges and for all types of melanoma, e.g., in thin primary melanoma lesions the WHO study investigated melanoma patients not thicker than 2 mm with a resection margin of 1 cm, while the Intergroup study took as conservative arm a margin of 2 cm for patients with a primary disease of 1–4 mm thickness. This difference, even if does not appear of great importance, may determine a major difficulty in repairing the wound in some circumstances, like the distal part of limbs or the head and neck area. The guideline followed at the European Organization for Research and Treatment of Cancer Melanoma Group (EORTC MG) and the WHO Melanoma Program 1997 meeting proposed a margin of 1 cm up to 2 mm Breslow, while a 2 cm margin is reserved for thicker lesions. Nevertheless, a margin of 1 cm can be proposed independently to the thickness if a wider surgical excision could be functionally or cosmetically deforming. This proposal should not be taken into consideration if a 2 cm margin would be of significant prognostic value, so in the absence of a scientifically determined indication for a margin of 2 cm around a primary lesion, we always suggest a conservative approach of 1 cm, which permits a primary closure in fairly all situations. We can state that a 1 cm resection margin is more frequently followed in Europe, while the larger margin is generally proposed in the USA [98,99,100].
